# Skip Fusion With Sacral-Alar-Iliac Screw Fixation for Pelvic Ring and Lumbar Fractures: A Case Report

**DOI:** 10.7759/cureus.50022

**Published:** 2023-12-06

**Authors:** Ryota Kimura, Yuji Kasukawa, Michio Hongo, Daisuke Kudo, Motoki Mita, Koji Nozaka, Naohisa Miyakoshi

**Affiliations:** 1 Orthopaedic Surgery, Akita University Graduate School of Medicine, Akita, JPN

**Keywords:** sacral–alar–iliac screw, range of motion, skip fusion, lumbar fracture, pelvic ring fracture

## Abstract

Pelvic ring fractures are associated with high-energy trauma and high mortality owing to critical blood loss and concomitant injuries. If there is a concurrent lumbar fracture, the postoperative range of motion will be limited owing to the increased fusion range. Here, we report a case in which skip fusion with sacral-alar-iliac screw fixation was effective as a minimally invasive procedure for treating pelvic ring and lumbar fractures.

## Introduction

Pelvic ring fractures account for 3% of all skeletal fractures [1] and are associated with a high mortality rate. Various techniques have been used to fix pelvic ring fractures. Recent studies have shown lower infection rates (2.9% to 7.1% with the posterior approach); however, concerns remain [2]. Concerns regarding infection and wound necrosis have, in part, led to increased interest in closed reduction and percutaneous fixation for treating these injuries [2]. In addition, when a pelvic ring fracture is concurrent with a lumbar fracture, the range of motion is limited postoperatively because of the longer fusion range. In this study, we report a case in which skip fusion with sacral-alar-iliac screw fixation (SAIFIX) was effective as a minimally invasive procedure for treating pelvic ring and lumbar fractures.

## Case presentation

**Figure 1:**
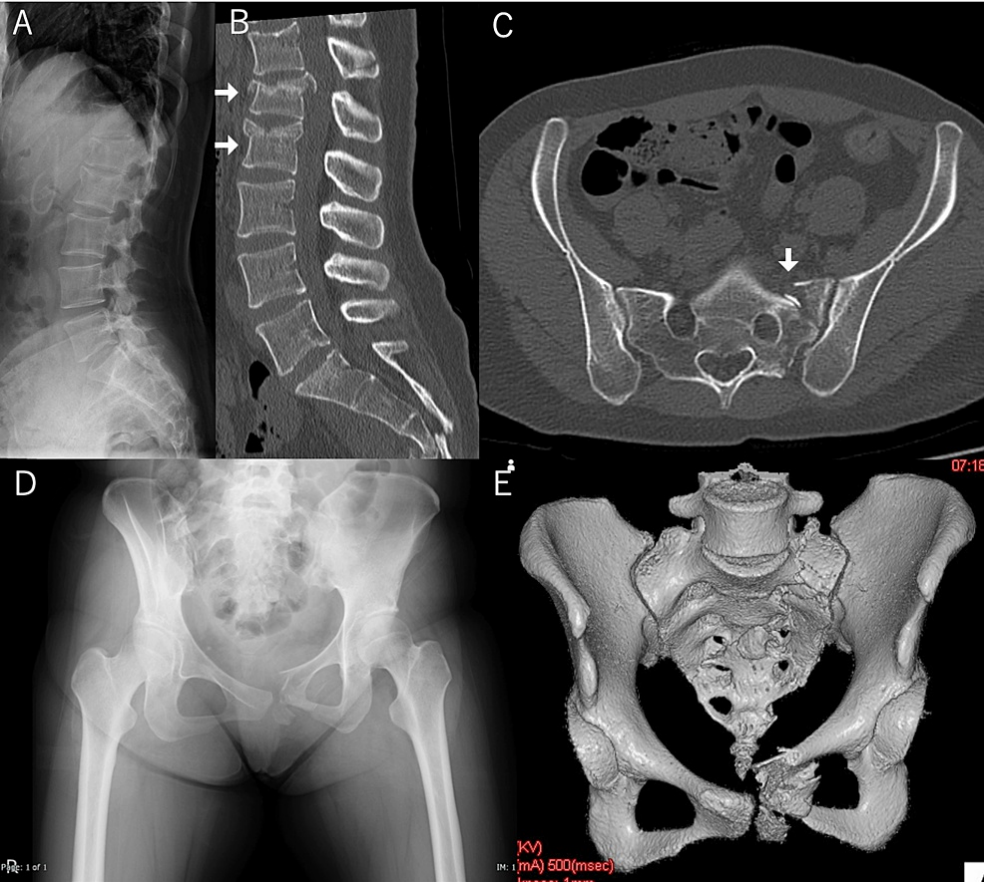
Findings at the time of transport to our hospital. (A) Lateral X-ray view shows fracture of the L1–2 vertebrae. (B) CT sagittal view of the spine shows the burst fracture of the L1–2 vertebrae and T12 spinous process (AO type classification; type B2) (white arrows). (C) CT axial view of the pelvis shows a left sacral fracture (white arrow). (D) Frontal X-ray shows a pelvic ring fracture. (E) Three-dimensional CT reconstruction shows a lateral compression type 1 pelvic ring fracture. CT, computed tomography

A 23-year-old female had postural instability due to sleeping pills for insomnia and an accidental fall from a window on the second floor. Computed tomography (CT) pan-scanning revealed an L1-2 burst fracture with a T12 spinous process fracture (AO classification [3]; type B2) and a pelvic ring fracture (lateral compression type 1 [4]) (Figures 1A-1E).

**Figure 2:**
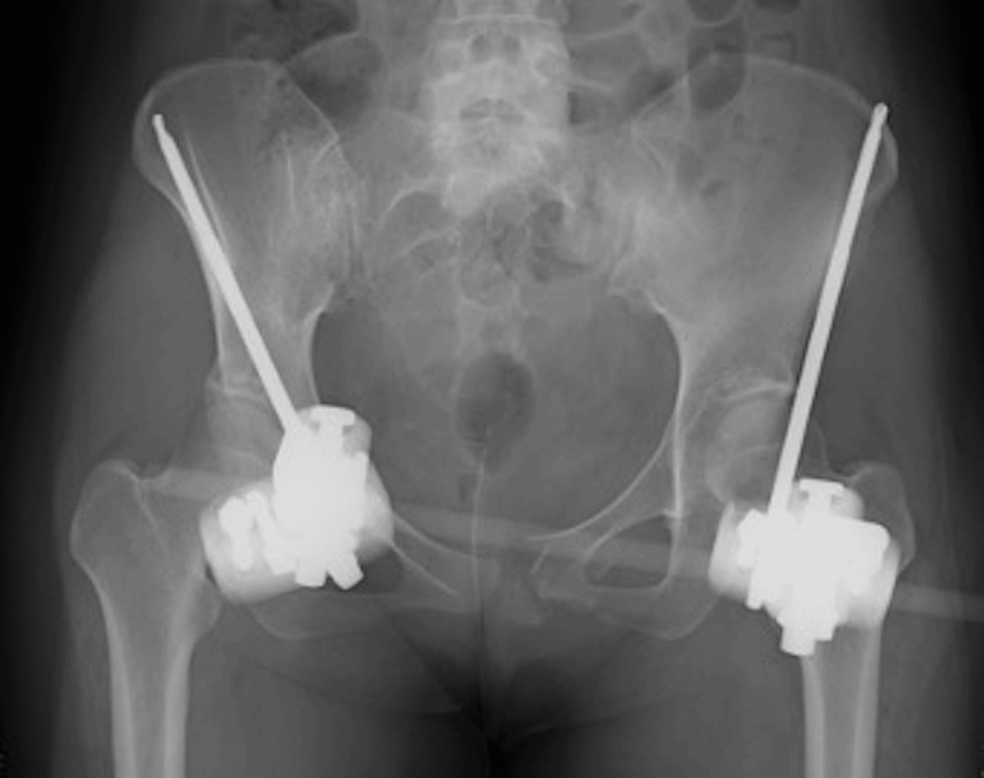
Image after external fixation surgery.

There were no neurological deficits and no hematuria retention; however, the patient was hemodynamically unstable. Her vital signs were as follows: blood pressure of 120/60 mmHg and a heart rate of 120 beats/min. On the same day, external pelvic fixation was performed to control the bleeding and stabilize the patient (Figure 2).

**Figure 3:**
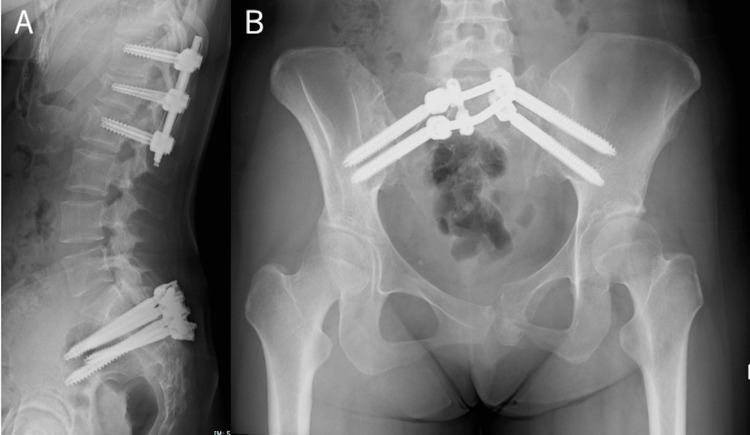
Postoperative imaging findings. (A) Lateral X-ray view after the surgery; posterior fixation was performed at the T12–L2 level and pelvis. (B) Frontal X-ray view of the pelvis after the surgery; the pubic symphysis is mildly reduced.

Evaluation under anesthesia revealed rotational instability of the pelvis. Her general condition stabilized at the intensive care unit, and on the fifth day postinjury, we performed posterior stabilization (T12-L2) with a percutaneous pedicle screw without a bone graft and SAIFIX, which involved posterior pelvic fixation with dual sacral-alar-iliac (SAI) screws connected to a transverse connector (Figure 3).

**Figure 4:**
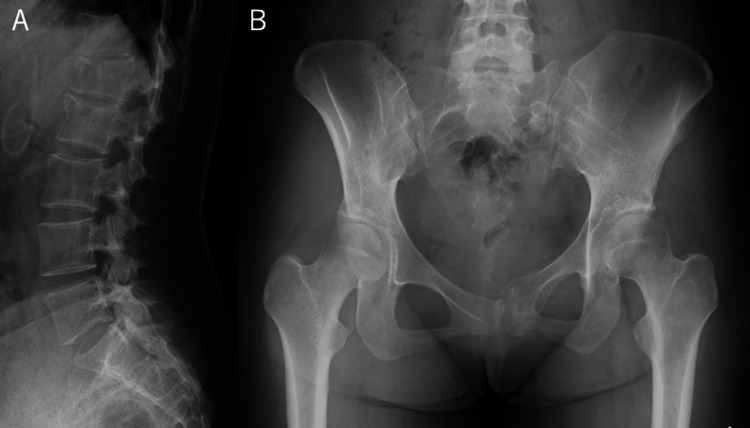
Imaging findings after implant removal. (A) Lateral X-ray view after implant removal. (B) Frontal X-ray view after implant removal.

Postoperative evaluation under anesthesia showed that the pubic symphysis fracture was stable, and the procedure was completed without additional anterior surgery. The patient began walking with full weight bearing the day after surgery. Two weeks after surgery, she was able to walk stably using a walker and was transferred to another hospital for rehabilitation. Three months postoperatively, the patient experienced no pain and was able to live independently in daily life. Lumbar flexion was slightly limited, with a finger-to-floor distance of 10 cm, but there were no limitations in activities of daily living (ADL). The patient's progress was good, and CT confirmed bony fusion. With a strong desire to return to work as soon as possible and after discussion with the patient, the implant was removed six months postoperatively (Figure 4).

She returned to work (as a nurse) seven months after the fixation surgery and one month after implant removal. Two years after surgery, she was able to continue working without pain.

## Discussion

SAI screws are used as pelvic anchors in adult spinal deformity surgery [5]. SAI screws are simple to insert yet effective as an anchor because it penetrates the tricortical bone and provides a strong fixation force owing to the long penetration of the screw [5]. One report indicated that SAI screws fixation for unstable pelvic ring fractures reduced blood loss compared to trans-iliac plate fixation [6]. Dual SAI screws [7] have been used as strong anchors for lumbosacropelvic fusion. The stability of SAI screws is stronger than that of iliac screws, sacroiliac screws, and transsacral-transiliac screws [8,9]. The use of unilateral dual SAI for pelvic insufficiency fractures was reported in 2013 [10]. In unstable pelvic fractures that do not require open reduction, we have used a fixation technique termed “SAIFIX” in which these dual SAI screws are interconnected to provide stability to the posterior pelvic ring. Indications for this method include posterior unstable fractures (AO types B and C) among pelvic ring fractures [6]. If the postoperative evaluation under anesthesia shows no instability of the anterior element, we consider the addition of anterior fixation unnecessary. The SAIFIX technique is minimally invasive, with bilateral screws inserted through a 5-cm longitudinal median incision. The rods can be connected to each other using a transverse connector to provide a stronger fixation. The screw heads and rods are located deeper than the posterior superior iliac spine, allowing for a low profile without touching the implant from the body surface. This technique offers several advantages. First, this method preserves the range of motion of the lumbosacral spine. Second, it allows posterior fixation with anterior external fixation during surgery, and screws can be inserted without iliac bone resection. Third, the SAI screws are greater in thickness and deeper relative to the skin compared to iliac screws. This may provide desirable clinical advantages in terms of the diameter of the selected screws and reduced protrusion [8].

In the case of pelvic fractures concurrent with lumbar vertebral fractures, skip fusion may be performed to reduce the restriction of the range of motion until the implant is removed. Skip fusion has been reported to have the potential to prevent adjacent intervertebral disease [11]. Longer-range fusion with spinopelvic fusion carries the risk of ADL impairment owing to limited spinal range of motion [12]. Patients who underwent one- or two-level fusion reported no serious limitations in most ADL, whereas patients who underwent three- or four-level fusion reported more limitations related to postoperative lumbar stiffness [13]. In this case, the range of fixation was limited to two-level fusion, and the postoperative ADL was maintained, which is considered an advantage of skip fusion.

This is the first report of skip fusion using SAIFIX for lumbar and pelvic ring fractures. Although further case reports and comparative studies using other surgical techniques are required, SAIFIX may be an effective treatment option for unstable pelvic ring fractures.

## Conclusions

Here, we report a case of skip fusion with SAIFIX for pelvic ring and lumbar fractures. This method maintains the range of motion of the lumbar and sacral spines and reduces surgical invasiveness. Therefore, comparative studies using other methods are required for further verification.
